# Reconsidering the roots, structure, and implications of gambling motives: An integrative approach

**DOI:** 10.1371/journal.pone.0212695

**Published:** 2019-02-22

**Authors:** Juan R. Barrada, Juan F. Navas, Cristian M. Ruiz de Lara, Joël Billieux, Gaëtan Devos, José C. Perales

**Affiliations:** 1 Departamento de Psicología y Sociología, Universidad de Zaragoza, Teruel, Spain; 2 Mind, Brain and Behavior Research Center (CIMCYC), University of Granada, Granada, Spain; 3 Addictive and Compulsive Behaviours Lab, Institute for Health and Behaviour, University of Luxembourg, Esch-sur-Alzette, Luxembourg; 4 Centre for Excessive Gambling, Lausanne University Hospital, Lausanne, Switzerland; 5 Laboratory for Experimental Psychopathology (LEP), Psychological Sciences Research Institute, Université catholique de Louvain, Louvain-la-Neuve, Belgium; 6 Service Universitaire d'Addictologie de Lyon (SUAL), CH Le Vinatier, Bron, France; 7 Scientific Research and Publication Cell (CRPS), Le Beau Vallon, Namur, Belgium; Ariel University, ISRAEL

## Abstract

**Rationale and method:**

Accurately identifying motives to gamble is crucial in the functional analysis of gambling behavior. In this study, a data-driven approach was followed to clarify the factor structure underlying a pool of motives for gambling, selected from the *Gambling Motives Questionnaire–Financial* (GMQ-F), and the *Reasons for Gambling Questionnaire* (RGQ), in a sample of regular problem and non-problem gamblers. Additionally, the role of gambling motives in the relationship between root behavioral activation/inhibition systems (BIS/BAS) and gambling severity, frequency, and preferences was explored using structural equation modelling (SEM).

**Results and conclusions:**

The present study identified Social, Financial, and Fun/thrill-related gambling motives factors, but also a fourth factor in which some positive and negative reinforcement-based motives were grouped into a single and broader Affect regulation factor. This Affect regulation factor shared variance both with BIS and BAS-related measures, and was the only direct predictor of disordered gambling symptoms. The Fun/thrill factor was directly related to frequency of participation in high-arousal, skill-based games, and all factors were related to participation in lower-arousal, chance games (with Social motives *negatively* predicting both participation in the latter and total severity). In the SEM model, measures of BIS/BAS sensitivity were connected to gambling behavior only through gambling motives. Based on measures of items’ specificity, a shortened Spanish scale (the brief Gambling Motives Inventory, bGMI) is proposed to assess gambling motives in accordance with the observed 4-factor structure.

## Introduction

Although it is often believed that gambling is mainly driven by financial motives, research has shown that gambling behaviors serve many other purposes. People gamble as a pastime, to have fun, for thrills, to share time or compete with others, to improve their abilities, to avoid boredom, to mitigate negative repetitive or intrusive thoughts, to curb cravings, or to relieve poor moods. Importantly, the effect of these motives is not mediated by expectancies about the potential monetary outcomes of gambling [1].

More specifically, gambling motives contribute to shaping gambling patterns by biasing risk-benefit weighting [2] and by interacting with other factors involved in disordered gambling, such as impulsivity and gambling preferences [3, 4, 5, 6]. Accordingly, identifying and assessing motives is a crucial component of functional analysis of problematic gambling behavior, and a necessary step for targeted interventions [5, 7].

Research has consistently shown that individuals suffering from Gambling Disorder (GD) are prone to gamble to manage negative affect, which is consistent with the hypothesis that gambling activities are partially maintained by negative reinforcement-related processes [8]. Thus, for rather transparent reasons, coping motives are associated with severity of gambling disorder (GD) symptoms [9, 10], with a speeded transition from recreational to disordered gambling [11, 12], and with comorbidities in the affective and impulse-control domains [13, 14, 15].

The link between positive reinforcement and disordered gambling is more complex. For instance, a recent study [16] reported gamblers playing at least once a week display more enhancement motives (gambling for fun or excitement), social motives (gambling to socialize, as part of social activities, to escape loneliness, or to build social connections) and financial motives (gambling to make money) than those playing less than once a week (see also [17]), but none of these motives discriminated between treatment-seeking problem gamblers and regular non-problem gamblers. Yet, complementary evidence shows that positively reinforcing motives, and particularly enhancement, directly correlate with signs of risky or problem gambling, such as intensity and time spent in gambling [18, 19], number of gambling activities [20], gambling-related cognitive distortions [5], and emotion-laden impulsivity [16, 21]. These findings are consistent with research suggesting that motivation to boost euphoria, thrill, or novelty is related to positive emotion-driven impulsivity, some forms of emotion dysregulation, and risk taking [22, 23, 24]. Conversely, evidence regarding social motives remains inconclusive, with reports of positive associations between social motives and problem gambling [17, 19], no association [25, 26], or even negative associations [27].

A suggested way to explore the soundness of the distinction between positively and negatively reinforcing motives has been to explore their relationships with the constructs of Gray’s psychobiological model of personality [28, 29]: the behavioral activation system (BAS) and the behavioral inhibition systems (BIS). BAS hyperactivity (reward sensitivity) is linked to extraversion and responsiveness to appetitive rewards and conditioned cues, whereas BIS hyperactivity (punishment sensitivity) is linked to neuroticism, sensitivity to aversive outcomes and threat cues, withdrawal, and avoidance [30, 31]. Accordingly, a number of studies have shown that BIS/BAS scores predict the proneness to endorse positive and negative gambling motives [32, 33].

However, evidence regarding BIS/BAS links with problem gambling remains inconclusive. Reward hypersensitivity, on the one hand, has been shown to predict risky, potentially problematic gambling [34], but BAS hyporesponsiveness has also been linked to gambling severity [35]. Similarly, punishment sensitivity has been observed to contribute to negative affect dysregulation and gambling severity [36], but also to protect gamblers from exposing themselves to gambling activities [34, 37]. These seemingly opposite effects [37, 38, 39] suggest that the relationships between BIS/BAS measures and gambling behavior are more complex than initially hypothesized. For example, a study [40] explored the relationships between the BIS/BAS constructs and addictive behaviors, and found differential associations between separate reward-related traits (e.g., Drive and Fun seeking) and gambling, alcohol use, and tobacco use. Importantly, these differential effects are compatible with the hypothesis that reward sensitivity could partly underlie individual differences in the subjective value allocated to different types of positive-reinforcement-related motives (e.g., fun, excitement, or joy seeking).

To our knowledge, the hypothesis that specific motives factors mediate the relationship between BIS/BAS-related dimensions and gambling behavior has been tested in two previous studies. First, Wardell et al. [33] formulated a model in which gambling motives mediated the relationship of reward and punishment sensitivity with gambling frequency and symptoms of disordered gambling. This model yielded an excellent fit, but the mediational model was not tested against any alternative model assuming direct links between reward/punishment sensitivity and gambling. Second, Sztainert et al. [32] investigated the potential mediation of gambling motives in the relationship between reward sensitivity and treatment seeking in problem gamblers. In this study, the association between reward sensitivity and willingness to seek treatment was observed to depend on social motives. However, neither punishment sensitivity, nor other aspects of gambling-related behavior were considered.

### Assessment of gambling motives

A useful factorization of gambling motives must be sufficiently detailed to differentiate between sources of reinforcement beyond their valence (positive vs. negative). At the same time, it must be compact enough to group similar motives together, and account for substantial shares of variability in other clinically or theoretically relevant constructs. Presently, the two most widely accepted methods of assessing gambling motives are the Gambling Motives Questionnaire-Financial (GMQ-F) [41]–based on the GMQ [10]–and the Reasons for Gambling Questionnaire (RGQ) [20, 21, 42]. These two instruments are aligned with recent theoretical models of gambling motives [43, 44, 45].

The GMQ-F includes four dimensions: Enhancement, Coping, Social, and Financial motives (see the first and second column from Table 1 for item correspondences of each factor). The RGQ includes these four, plus a Recreation factor (please note, however, that the two scales present little overlap in specific items; Table 1, columns 1 and 3). Among these, Enhancement and Coping motives (from the GMQ-F) are associated with problematic and disordered gambling [16]. The RGQ validation study did not include a sufficiently large number of problem gamblers to attain enough observations in high levels of the severity continuum. Nevertheless, those gamblers who showed a mixed pattern of online and land-based gambling, also showed higher scores on enhancement, recreational and financial motives than exclusively offline gamblers.

**Table 1:** Items assignment in the original scales, descriptives, and loadings in factor analyses.

								Full Motives pool				bGMI			
	GMQ-F	RGQ	*M*	*SD*	*Sk*	*K*	*Per*	FUN	AFF	SOC	FIN	FUN	AFF	SOC	FIN
*1. Because I'm worried about not winning if I don't play		FIN	0.26	0.54	2.4	6.6	22.2	–.10	.26	.22	**.48**	––	––	––	––
*2. To compete with others (e.g., bookmaker, other gamblers)		ENH	0.34	0.68	2.1	4.0	24.6	.15	.21	**.34**	–.17	––	––	––	––
*3. Because I enjoy thinking about what I would do if I won a jackpot	FIN		1.24	1.01	0.5	-0.8	74.4	.02	.02	**.38**	**.57**	––	––	––	––
4. To earn money	FIN		1.04	1.03	0.6	-0.8	62.1	–.02	.20	–.01	**.74**	.02	.20	-.09	**.69**
5. As a hobby or past-time^a^		REC	0.97	1.09	0.7	-0.9	53.5	**.87**	–.01	.00	–.05	**.81**	-.04	.06	-.12
*6. To escape boredom or to fill my time		REC	0.64	0.90	1.2	0.3	39.4	**.63**	**.34**	.02	–.19	––	––	––	––
7. Because it’s fun	ENH	REC	0.91	0.93	0.6	-0.8	57.1	**.77**	.05	.25	–.02	**.83**	.02	.27	-.02
*8. Because it's something that I do with my friends or family		SOC	0.91	0.98	0.9	-0.3	57.6	**.46**	**–.35**	**.65**	.05	––	––	––	––
9. Because I feel more self-confident or sure of myself	COP		0.19	0.51	3.1	10.6	14.8	.27	**.58**	–.02	.14	**.50**	**.45**	-.22	.01
10. Because it’s what most of my friends do when they get together	SOC		0.27	0.61	2.5	6.1	19.7	–.07	.21	**.68**	–.14	-.03	**.39**	**.54**	-.01
*11. Because of the sense of achievement when I win		ENH	0.52	0.81	1.5	1.4	35.0	.09	**.35**	**.30**	**.30**	––	––	––	––
12. Because it’s exciting	ENH	ENH	1.05	1.01	0.5	-0.9	62.1	**.61**	.23	.14	.24	**.70**	.18	.09	.21
13. To relax		REC	0.33	0.68	2.1	3.6	22.7	.19	**.84**	–.15	–.02	.19	**.79**	-.09	-.07
*14. For the mental challenge or to learn about the game or activity		ENH	0.53	0.85	1.6	1.5	34.0	**.44**	.12	**.38**	–.03	––	––	––	––
15. Because winning would change my lifestyle	FIN		0.58	0.87	1.5	1.4	38.4	–.06	–.13	.22	**.75**	-.07	-.05	.22	**.79**
16. To cheer me up when I am in a bad mood	COP		0.27	0.59	2.4	5.7	20.2	–.03	**.88**	.07	.00	-.02	**.89**	.12	.01
17. To forget my worries	COP		0.36	0.77	2.3	4.4	23.2	–.16	**1.01**	.02	.01	-.15	**1.02**	.03	.00
18. To win money / To make money ^c^	FIN	FIN	1.29	1.09	0.3	-1.2	70.0	.27	.01	–.11	**.97**	.24	-.01	-.08	**.96**
19. Because it helps when I'm feeling tense		COP	0.28	0.61	2.3	4.9	20.7	.02	**.89**	.08	–.05	.00	**.90**	.16	.01
*20. To be sociable ^a^	SOC	SOC	0.26	0.63	2.7	7.1	18.3	.04	.18	**.75**	**–.41**	––	––	––	––
21. Because it’s something I do on special occasions	SOC		0.72	0.89	1.1	0.3	48.8	.05	–.24	**.64**	.09	.10	-.10	**.51**	.24
22. Because it makes a social gathering more enjoyable ^c^	SOC		0.38	0.72	2.0	3.3	25.7	.22	.00	**.75**	–.26	.14	.18	**.82**	-.04
*23. To impress other people		COP	0.09	0.36	4.3	18.5	6.4	–.20	**.48**	**.48**	.04	––	––	––	––
24. Because it makes me feel good	ENH		0.39	0.71	1.9	3.4	28.1	.06	**.77**	.13	.06	.18	**.75**	.03	.06
25. Because I like the feeling	ENH		0.39	0.71	1.9	3.4	28.1	.16	**.66**	.16	.15	**.39**	**.60**	-.03	.09
26. For the chance of winning big money ^b^		FIN	1.10	1.07	0.5	-1.0	62.1	.07	.06	.01	**.82**	-.03	.14	.04	**.82**
27. Because it helps when I am feeling nervous or depressed ^b^	COP		0.30	0.71	2.6	6.1	18.7	.19	**.88**	–.22	.00	.12	**.82**	-.03	-.05
Interfactor correlations							FUN								
							AFF	.50				.54			
							SOC	.44	.46			.33	.17		
							FIN	–.01	.15	.12		.08	.17	-.16	

Note: GMQ-F = Gambling Motives Questionnaire—Financial; RGQ = Reasons for Gambling Questionnaire; bGMI = brief Gambling Motives Inventory; *M* = mean*; SD* = standard deviation*; Sk* = skewness*; K* = kurtosis. *Per* = percentage of respondents with a response different from *never*; ENH = Enhancement; COP = Coping; SOC = Social; FIN = Financial; REC = Recreation; FUN = Fun/thrill; AFF = Affect regulation.

Items with an asterisk correspond to items not included in shortened version of the scale (bGMI). Bold loadings indicate loadings over |0.30|. Underlined loadings indicate maximum loading among factors.

^a^*n* = 202

^b^*n* = 182

^c^ Slightly different wordings are used in GMQ-F and RGQ.

* Items not included in the bGMI.

In some cases, instruments have been purposely tailored for the aims of the study, following a data-driven approach [15] (see also [1]). The gambling motivation study with the largest sample to date (4,125 Internet gamblers) [15] selected a number of motives from previous works [10, 45, 46, 47, 48], and performed a principal component analysis (PCA) in order to isolate main constituents. The PCA yielded three factors: Money (equivalent to financial motives in other instruments), Enjoyment, and Mood Regulation. This last factor included a variety of motives like coping, affect upregulation, escape from routine and boredom, and dealing with urges. Moreover, this factor most clearly discriminated between problem and non-problem gamblers. Relevantly, both people displaying very high (hypomania) or low mood (depression) presented higher scores in this last dimension.

### Aims and hypotheses

The first aim of this study was to clarify the factor structure underlying the gambling motives expressed in a pool of items (from the GMQ-F and the RGQ), in a sample of regular pathological and non-pathological gamblers. This integrative and exploratory approach was adopted, first, to avoid results that could be driven by the specific motives included in a single instrument; and, second, to keep it data-driven and as independent as possible of specific *a priori* theoretical frameworks. Indeed, previous studies have shown that the best-fitting factor structure can slightly vary across cultures and settings [20], and, therefore confirmatory approaches can force data to fit into preconceived models. The selection of these two specific scales was aimed to attain two goals: (1) to cover most specific motives described in previous literature, and (2) to accrue a sufficiently large number of items with different wordings to further select the ones with better psychometric properties.

The second aim was to reduce the number of items to a more manageable set, by selecting the items with higher and more specific factor loadings. This reduced set with better psychometric properties will be used for further analyses, and, additionally, will be evaluated in terms of usefulness as a brief scale for research and clinical practice.

And thirdly, we intended to explore the role of gambling motives in the relationship of BAS and BIS with gambling severity and frequency. As noted above, BAS and BIS are responsible for general proneness to respond to the prospect of appetitive and aversive events. Endorsement of specific positive motives will thus be more widespread in gamblers with high reward sensitivity scores, whereas negative ones will be more prevalent in punishment-sensitive gamblers. Our specific hypothesis here is that gambling motives are contextually sensitive; they do not depend exclusively on reward and punishment sensitivity, but also on the history of interaction between the gambler and its environment. In other words, basic BIS/BAS traits are expressed in different patterns of gambling motives in people with different learning histories, and these motives, in turn, contribute to shape specific gambling behaviors. Indeed, we assume that the inconsistent findings observed in previous studies can be (at least partly) due to the fact they did not consider gambling motives when analyzing the relationships between BIS/BAS and gambling. Accordingly, BIS/BAS measures will be used in structural equation modeling (SEM) as input variables, motives as mediating variables, and gambling severity and frequency as output variables. For the assessment of gambling frequency, we will rely on the distinction between Type I (high arousal, skill-based) and Type II (lower-arousal, chance) games [49]. This will also allow us to assess how gambling motives moderate the relationship between overarching personality dimensions and gambling preferences.

As noted earlier, there has been a previous comparable attempt to test the mediational role of motives in the relationships between BIS/BAS measures and gambling severity and frequency [33]. The present study, however, is further designed to (1) test the mediational model against an alternative model in which BIS/BAS measures are allowed to predict gambling behavior independently of gambling motives; (2) assess gambling frequency for different types of games, in such a way that the relationship between gambling motives and gambling preferences can also be tested; and (3) factorize gambling motives using an integrative, data-driven methodology, so that it is not necessarily subject to previous models.

With these aims in mind, and assuming correspondence between the structure extracted by our exploratory factor analysis and the five factors identified in the original scales, our main hypotheses are stated as follows: (a) reward sensitivity is expected to uniquely contribute to positive (enhancement, recreation, financial, and social) motives, whereas punishment sensitivity is expected to contribute to coping motives; (b) both enhancement and coping motives are hypothesized to be associated with gambling severity, yet a more pronounced link is expected for coping; and, (c) enhancement motives will be predictably linked to frequency of participation in Type I games.

The possibility also exists that the resulting factorization does not exactly mirror the five-factor partition implied by the validation studies of the two original questionnaires. As noted above, the PCA analysis reported by Lloyd et al. [15], with items selected from various instruments, did not identify an Enhancement factor, segregated from coping. Instead, it yielded a more general Affect regulation factor that included items from both dimensions (whereas an enjoyment factor included social, amusement, and fun-related motives). If this were the case, mood regulation could be plausibly fueled by both reward and punishment sensitivity, and could also contribute, not only to gambling severity, but also to a higher frequency of participation in Type I games.

## Method

### Ethics statement

The procedure of this study complies with the ethical standards of the Helsinki Declaration of 1975, as revised in 2008, and was approved by Human Research Institutional Review Board of the University of Granada, as part of the GBrain 2 Project (Reference: PSI2017-85488-P, IRB approval number 406/CEIH/2017). All participants were informed about the study’s objectives and provided informed consent.

### Participants and procedure

Two hundred and three gamblers took part in this study. Sociodemographic data and gambling characteristics are displayed in Table 2. The sample included 25 treatment-seeking patients with GD recruited from an outpatient clinical center (AGRAJER, *Asociación Granadina de Jugadores de Azar en Rehabilitación*). The rest of the sample was formed by occasional or regular gamblers, who were recruited by a snowball sampling method, using Internet posting, researcher’s social media, and advertisement in the campus of the University of Granada. Inclusion criteria for the entire sample were (1) being older than 18 years old, (2) fluent in Spanish, and (3) having ever gambled–whatever the amount of money and type of gambling. This latter criterion was established based on the idea that gambling motives are present as soon as there exists any detectable gambling activity. Nevertheless, only 3 participants in our sample had ever gambled, but did so less than once a year, and 8 gambled in only one modality between 1 and 5 times per year. Based on the SOGS questionnaire, 103 participants had never experienced any significant negative consequence due to gambling.

**Table 2:** Descriptive data of the sample.

	*Total sample* *(n = 203)*		*Disordered gamblers* ^*a*^ *(n = 31)*^*b*^		*Non-disordered gamblers* *(n = 168)*	
	*Mean (SD)*		*Mean (SD)*		*Mean (SD)*	
Age	33.84 (14.05)		34.84 (11.66)		33.75 (14.48)	
Sex	69 females		1 female		67 females	
Years of education	15.33 (3.91)		13.68 (3.35)		15.62 (3.89)	
Monthly income in Euros	1935.61 (827.79)		2016.13 (826.48)		1913.11 (832.43)	
Gambling severity*	2.00 (3.57)		9.45 (3.35)		0.62 (0.91)	
Gambling frequency**	*n = 199*		*n = 31*		*n = 164*	
	Type I	Type II	Type I	Type II	Type I	Type II
Daily	11	13	7	11	4	1
2–6 times a week	14	8	7	5	6	3
Weekly	11	38	5	5	6	33
2–3 times a month	11	24	2	3	9	21
About once a month	11	21	0	4	10	15
6–11 times a year	11	12	1	1	10	11
1–5 times a year	36	63	2	1	33	61
Never	94	19	7	1	86	18
Mean duration per typical day in minutes	113.46 (189.46)	41.72 (88.44)	227.84 (249.00)	154.37 (160.31)	90.95 (169.57)	20.83 (43.53)
Mean amount of money per typical day in Euros	55.84 (347.70)	36.57 (118.44)	299.69 (842.65)	177.09 (259.37)	10.77 (52.59)	10.80 (14.44)
Maximum amount in a single day in Euros	222.90 (1548.77)	137.05 (585.88)	1296.68 (3785.26)	753.24 (1337.97)	24.73 (129.91)	23.64 (44.15)

^a^ Gamblers with a score equal or higher than 5 in SOGS were categorized as disordered gamblers [87].

^b^ 25 disordered gamblers were in treatment.

*Gambling severity as assessed by SOGS.

** Gambling frequency by game modality (ad-hoc survey).

Type I: Cards, casino games, skills and sports bets; Type II: Lotteries, pools, bingo, and slot machines. Apparent discrepancies are due to the following missing data: Age = 7, sex = 7, year of educations = 5, monthly income = 5, Gambling severity = 4; Gambling frequency = 4 (Gambling frequency Type II = 5).

This sampling method was intended to include gamblers across the whole severity continuum, and to ensure a sufficient number of observations at pathological levels. This method, however, despite being pervasive in the relevant literature, does not ensure representativeness. This lack of representativeness, along with the relatively small sample size, could have generated inferential problems that are identified and discussed in the closing section of the present article.

One hundred and fifty-nine participants were assessed with paper-and-pencil instruments; 63 of them were provided with assessment booklets to complete the questionnaire at home. The remaining 44 participants completed the questionnaires by means of an online survey platform. All participants were debriefed on the aims and instructions, either personally (e.g., prior to the delivery of the assessment booklets) or by email. In the latter case, participants were asked if they had read and understood the aims and instructions, and gave explicit consent to participate, before being given permission to access the survey platform. The remaining participants signed a written informed consent. Personal assessments and debriefings were performed by examiners with a psychology degree, under the supervision of a seasoned researcher with seven years of experience in psychological assessment.

The whole assessment protocol comprised several self-report measures, some of which were not directly related to the aims of the present study. In addition, 21 of the 25 patients with GD were selected to participate in a larger assessment protocol (the rest of which was programmed for a different session), including neuropsychological tasks and fMRI measures that will be also presented in future reports. Data were collected between October 2015 and October 2017.

### Measures

#### Gambling motives questionnaires

The two gambling motives questionnaires used in the present study were the Reasons for Gambling Questionnaire (RGQ) [42] and the Gambling Motives Questionnaire–Financial (GMQ-F) [41]. The GMQ-F includes Enhancement (e.g., “*Because it is exciting*”), Coping (e.g., “*Because it helps me when I feel anxious or depressed*”), Social (e.g., “*Because it is something I do on special occasions*”), and Financial (e.g., “*To make money*”) motives. The RGQ includes the same gambling motives, plus a Recreation factor (e.g., “*It is something I do as a hobby or a pastime*”).

Spanish item versions were worded according to a standard back-translation procedure. Items from the two questionnaires were presented in the original order, and each items scored in Likert-type scale ranging from 0 = *never/almost never* to 3 = *always/almost always*. When pairs of items belonging to different questionnaires (GMQ-F or RGQ) were judged as semantically identical by the translator and two of the authors (CMRL and JCP), one of them was removed. It is worth noting that, for only one of these pairs, the items were not assigned to the same factor in the two original questionnaires (“*because it is fun*” is postulated to reflect Recreation in the RGQ and Enhancement in the GMQ-F). The four items that were judged to be identical in the two scales are numbered as 7, 12, 18, and 20 in the leftmost column of Table 1.

As displayed in Table 1, the final pool consisted of 27 items (column 1). The table also specifies their assigned factor in their respective original instruments (columns 2 and 3), and their descriptives (columns 4–8). Due to a problem in the printable version of some questionnaires, two items were lost for 21 participants (all of whom were non-problem gamblers). As noted in the note of Table 1, *n* for these items was 182. There were also three lost responses for items 5, 20 and 22 (so *n* for these items was 202).

#### South Oaks Gambling Screen (SOGS) [50]

The SOGS assesses symptoms of disordered gambling. It also provides measures of dependence and debt accrual. In addition, it has shown adequate convergent validity with the diagnosis of GD, following DSM-IV and DSM5 criteria (.93 correlations in both cases) [51]. The Spanish version used in this study has shown good psychometric properties [52]. Cronbach’s alpha in the present sample was .92.

#### Gambling frequency by game modality

Ad-hoc items were generated in order to assess participation in 18 different game types [scratch cards, lotteries/pools, in-person card games in licensed venues, in-person card games in unlicensed venues, in-person card games in social or family occasions, online card games, in-person bingo, online bingo, slot machines (in person), slot machines (online), in-person casino games (excluding cards and slots), online casino games (excluding cards and slots), betting on one’s skills, online sports bets (excluding pools), in-person sports bets (excluding pools), stock markets, other online games, other in person games]. For each of these gambling activities, participants were asked whether they had played that game in the last 12 months. If they answered *no*, they were asked to skip that game, and proceed to the next one. When they answered *yes*, they were asked to report how frequently they played that game (1-between once and five times a year, 2-between 6 and 11 times a year, 3-about once a month, 4-twice or three times a month, 5-weekly, 6-between 2 and 6 times a week, 7-daily). Participants were also asked to report how many hours and minutes they spent playing each game in a typical day, how much money they spent in that game in a typical day, and what was the maximum amount they had spent in that game in a single day. Only frequency data were considered here for further analyses. For analyses, we used the dichotomy between Type I (cards, casino games, skills, and sports bets) and Type II (lotteries, pools, bingo, and slot machines), as described in a recent work [49]. Frequencies were simply summed across games in the same category to obtain Type I and Type II frequency scores for each participant.

#### Sensitivity to Punishment and Sensitivity to Reward Questionnaire (SPSRQ) [53]

This questionnaire assesses reward and punishment sensitivity. We used the shortened version from [30]. It consists 10 dichotomous items (yes/no) for each trait. Cronbach’s alpha in the present sample for Sensitivity to Punishment was .78 and for Sensitivity to Reward, .79.

### Statistical analysis

Statistical analyses were carried out in three consecutive steps. In the first step, descriptives were computed for all the items from the GMQ-F/RGQ: mean, standard deviation, skewness, kurtosis, and percentage of respondents with a response different from *never/almost never*.

In the second step, we evaluated the internal structure of the questionnaires. For gambling motives items, four and five-factors solutions were tested with an exploratory structural equation model approach (ESEM) [54]. As previously discussed, the number of factors to be extracted was uncertain, so the selection between the two solutions was strictly driven by model fit, and interpretability of the loading pattern.

In order to develop a usable tool, we selected the items that clearly loaded in a single factor. From the selected factorial solution, items for which (a) loadings in all the factors were below |.50|, or (b) more than a single loading was above |.30|, were dropped. Henceforth, we will refer to that selection of items as the *Brief Gambling Motives Inventory* (bGMI).

Both for the pool of motives and the SPSRQ, we compared the confirmatory factor analysis (CFA) models against ESEM. Although common CFA models are more parsimonious than ESEM (as no cross-loadings are specified), CFA results can distort inter-factor correlations and loading sizes [54, 55]. ESEM models are thus preferred over CFA ones when they yield better fits, when substantial cross-loadings exist, or when inter-factor correlations differ among solutions. For the SOGS, we only computed an ESEM (as for unidimensional scales CFA and ESEM results are equivalent). The internal structure of the gambling frequency survey was not tested due to the large proportion of items with floor effects, and the fact that the Type I/Type II categorization is justified by the abovementioned previous research.

In the third step, we modeled the responses to the items from all the questionnaires simultaneously. In a first *measurement* model, all the factors and the two gambling frequency scores were allowed to correlate with each other. The latent factors included in the model were SPSRQ Sensitivity to Punishment and Sensitivity to Reward, the motives factors extracted from the bGMI, and SOGS. As for all the questionnaires, the ESEM model was preferred over the CFA model (see Results section), and all the inter-item correlations of the different questionnaires were modeled using ESEM. In the measurement model, each questionnaire defined a separate set of factors, that is, although cross-loadings in the ESEM models were allowed within each questionnaire, cross-loading was not allowed between different questionnaires (i.e., Item 1 of the bGMI is allowed to load with all the factors of the bGMI ESEM set, but not with SPSRQ or SOGS factors). In a second, *structural* model, the motives factors were predicted by the SPSRQ factors, and motives factors predicted the SOGS factor and Type I and II gambling frequency scores, with the latent variables being modeled as in the measurement model. Thus, in the structural model, no direct relations between SPSRQ and gambling behavior were tested.

As the initial measurement model did not properly converge (probably due to its complexity and the relatively low sample size), we decided to create item parcels for simplification purposes [56]. To identify a model without convergence issues, it was needed to generate parcels only for SOGS items. Parceling was carried out using the one-dimension scale to avoid problems with items simultaneously tapping onto more than one dimension. Thus, three parcels were created with SOGS items, by averaging scores from individual items. Items were assigned to parcels based on their item number (the first item to the first parcel, the second item to the second parcel, the third item to the third parcel, the fourth item to the first parcel, and so on).

Importantly, these models were fitted using the motives items characterized by the best psychometric properties (i.e., items with loadings below |.50| for all factors, or with more than a single loading above |.30|, were dropped). This decision was made to avoid SEM interpretability to be limited by sample size, as, although an *n* = 200 sample size is considered large enough for the type of estimator used here [57], it could fall short when the items and measures included in the model present low reliability.

Goodness of fit of all the derived models was assessed with the common cut-off values for the fit indices [58]: CFI and TLI with values greater than .95 and RMSEA less than .06 are indicative of a satisfactory fit. For all the models, the WLSMV estimator was used, in order to maintain the categorical nature of the responses [59]. For all the factor models we interpreted the standardized solution (STDYX solution in *Mplus*).

ESEM and CFA models were estimated with *Mplus* 7.4 [60]. The rest of the analyses were performed with R 3.5.0 [61]. We used the package *MplusAutomation* version 0.7 [62]. Reliabilities of the bGMI dimensions were computed with Cronbach's alpha.

The open database and code files for these analyses are available at the Open Science Framework repository (https://osf.io/drfpu/).

## Results

### Descriptives of motive items

Descriptives for items in the 27-item pool are displayed in Table 1. The items presented low means (*M*_mean_ = 0.58, range [0.09, 1.26]), low standard deviations (*M*_SD_ = 0.79, range [0.36, 1.09]), positive skewness (*M*_Sk_ = 1.70, range [0.3, 4.3]), and a leptokurtic distribution (*M*_K_ = 3.17, range [–1.2, 18.5]). The proportion of respondents with responses different from *never/almost never* in each of the motives ranged from 6.4% (Item 23) to 74.4% (Item 3).

### Internal structure of the different instruments

#### Pooled motives list

We tested four and five-factor solutions for the 27 GMQ-F/RGQ items with an ESEM (Table 3). In both cases the model was satisfactory (four/five factors: CFI = .987/.993, TLI = .981/.989, RMSEA = .042/.032). The improvement in model fit of the five-factors solution over the four-factors solution was negligible (ΔCFI = .006, ΔTLI = .008, ΔRMSEA = –.010), and, in the five-factor solution, some factors had only one or two pure indicators (items with loading over |.50| and no cross-loading over |.30|). Consequently, the 4-factor solution was clearly preferable. The correlations among the different factors were in the range [.44, .50], except for the Financial factor (|*r*s| ≤ .15). Loadings and inter-factor correlations are displayed in Table 1. Despite the identifiability of the factors content, nine items were marked as problematic due to a low primary loading or relevant cross-loading.

**Table 3:** Goodness of fit indices for the different models.

Models	*n*	χ^2^	*Df*	*p*	CFI	TLI	RMSEA
M1. ESEM Motives pool 4F	203	336.4	249	.000	.987	.981	.042
M2. ESEM Motives pool 5F	203	272.0	226	.020	.993	.989	.032
M3. CFA bGMI 4F	203	237.9	129	.000	.980	.976	.064
M4. ESEM bGMI 4F	203	136.6	87	.001	.991	.984	.053
M5. CFA SPSRQ	196	217.4	169	.007	.960	.955	.038
M6. ESEM SPSRQ	196	148.8	151	.536	1.000	1.002	.000
M7. ESEM SOGS	199	181.0	152	.054	.996	.995	.031
M8. MEASUREMENT MODEL: ESEM bGMI 4F –ESEM SPSRQ–ESEM SOGS–FREQ	Non-positive definite latent variable covariance matrix						
M9. MEASUREMENT MODEL: ESEM bGMI 4F –ESEM SPSRQ–ESEM SOGS PARCELS–FREQ	203	853.7	766	.015	.983	.980	.024
M10. STRUCTURAL MODEL: ESEM bGMI 4F –ESEM SPSRQ–ESEM SOGS PARCELS–FREQ	203	861.9	772	.013	.983	.980	.024

Note: *df* = degrees of freedom; TLI = Tucker-Lewis index; CFI = comparative fit index; RMSEA = root mean square error of approximation; ESEM = exploratory structural equation modeling; CFA = confirmatory factor analysis. SPSRQ: Sensitivity to Punishment and Sensitivity to Reward Questionnaire. SOGS: South Oaks Gambling Screen. bGMI: brief Gambling Motives Inventory. FREQ: Gambling frequency per game ad hoc survey. PARCELS: Measurement model with item parcels (see text).

Although various labels were considered, the identifiable factors were finally named as Fun/thrill, Affect regulation, Social, and Financial. The third and fourth factors mostly matched the corresponding ones in the GMQ-F and the RGQ, so equivalent labels were used. However, our analyses yielded some non-trivial differences regarding the other factors (Enhancement, Recreational, and Coping in the RGQ and the GMQ-F). On the one hand, the GMQ-F included 4 enhancement motives (corresponding to items 7, 12, 24 and 25 in Table 1). In our analysis, two of these items (24 and 25) pooled together with Coping motives from both the GMQ-F and the RGQ, and with the motive “*To relax*” (which is classified as Recreational in RGQ). The two remaining items, corresponding to Enhancement in the GMQ-F (“*Because it’s fun*”, and “*Because it’s exciting*”) pooled with “*As a hobby or pastime*”. Of these latter three, “*Because it’s fun*” was labelled as Recreation in RGQ and Enhancement in GMQ-F, “*Because it’s exciting*” was labelled as Enhancement in both RGQ, and GMQ-F, and “*As a hobby or pastime*” was labelled as Recreation in RGQ (and is absent from GMQ-F). In other words, the GMQ-F Enhancement factor was not reproduced; some of its items pooled together with coping motives, and some others with RGQ Recreation motives. In view of this, neither Enhancement nor Recreation labels allow to describe the content of the items comprised by the first factor reported in Table 1. Accordingly, we decided to label this factor as Fun/thrill, as it seems to better capture the common semantic content of the items loading onto it.

The GMQ-F included four Coping motives. These four pooled together with “*To relax*” (Recreation in the RGQ), with “*Because it helps when I'm feeling tense*” (Coping in RGQ), and, as noted above, with “*Because it makes me feel good*”, and “*Because I like the feeling*” (Enhancement in GMQ-F). Thus, only five of the eight items distinctively loading onto that factor in our analysis had been previously categorized as Coping motives in either the RGQ or the GMQ-F. The other three items correspond to positive-reinforcement-related motives, and they had been categorized as either Enhancement or Recreational motives in previous studies. Given that coping is the customarily term used to specifically designate the regulation of negative affect, we selected the label Affect regulation, intended to denote, not only coping, but also positive affect upregulation.

Once problematic items were dropped, the shortened survey (henceforth the *brief Gambling Motives Inventory*, bGMI) consisted of 18 items. For this version, both a CFA and the ESEM fit are satisfactory (CFA/ESEM: CFI = .980/.991, TLI = .976/.984, RMSEA = .064/.053). We preferred the ESEM model for three reasons. The improvement in ESEM fit relative to CFA model was over |.010| for the indices; cross-loadings were as large as .45; and the mean unsigned correlation was .42 for the CFA model but .24 for the ESEM. In the bGMI, factor content closely matched the one of the full items pool. Finally, three items corresponded to the Fun/thrill dimension (Cronbach's α = .84), eight items to the Affect regulation dimension (α = .91), three items to the Social dimension (α = .56), and four to the Financial dimension (α = .85) (for the implications and potential solutions for the low reliability of the social dimension see the Discussion section below). Factor loadings for the 27-item pool (columns 9–12) and the reduced 18-item bGMI (columns 13–16) are reported in Table 1.

#### SPSRQ and SOGS

As the validation of these instruments is not the focus of the present study, we will only briefly comment that an ESEM model for the SPSRQ responses showed an appreciable improvement in fit over the CFA model (ΔCFI = .040, ΔTLI = .047, ΔRMSEA = –.038). For the SOGS, model fit was excellent. Thus, for all the considered scales, an ESEM approach was selected.

### Relations between constructs

#### Measurement model

As noted above, the initial model incorporating all items presented estimation problems: the latent variable covariance matrix (psi) is not positively definite. We thus created item parcels for the SOGS items. With this reduced model the model fit was very good (CFI = .983, TLI = .980, RMSEA = .024).

#### Structural model

**Fig 1:**
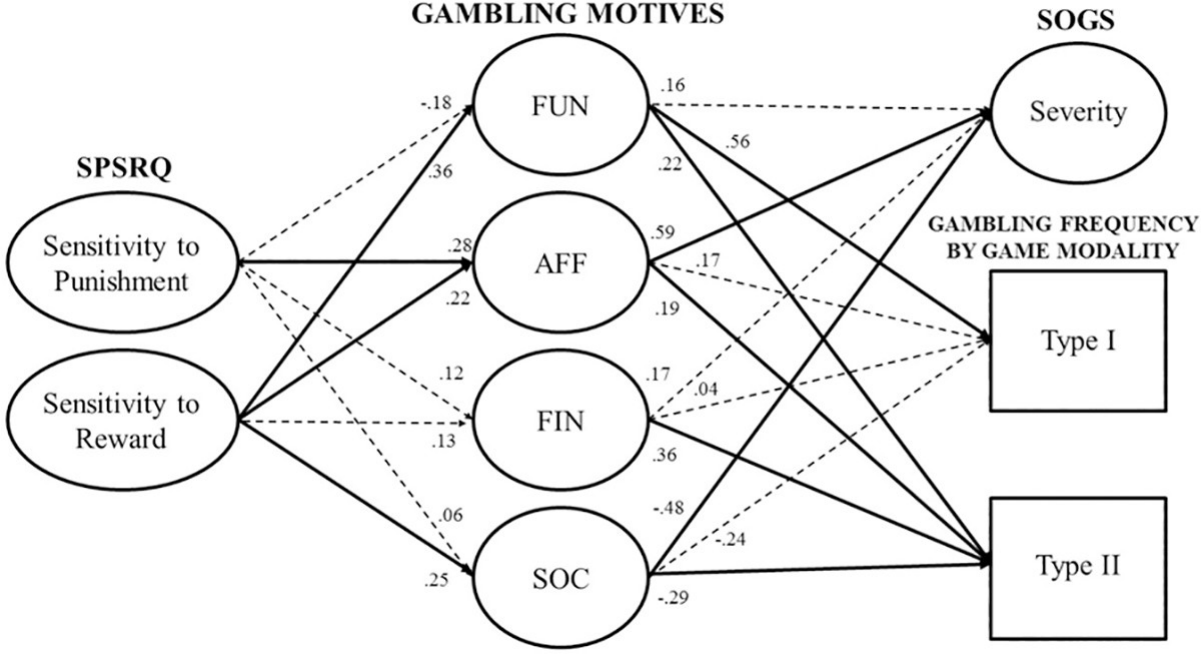
Structural Equation Modelling (SEM) of the relationships between the studied variables. Abbreviations: SPSRQ, Sensitivity to Punishment and Sensitivity to Reward Questionnaire; FUN, Fun/thrill; AFF, Affect regulation; FIN, Financial; and SOC, Social (i.e., gambling motives factors, as measured by the brief Gambling Motives Inventory [bGMI]); SOGS, South Oaks Gambling Screen (gambling severity). Note: Type I (high arousal, skill-based) and Type II (lower-arousal, chance) games, as measured by an ad-hoc gambling frequency survey. Solid lines correspond to statistically significant coefficients, *p* < .05. All nodes are latent variables, except for Type I and Type II frequencies (manifest variables). Dashed lines correspond to non-significant coefficients, *p* > .05.

Importantly, the model fit of the structural model was essentially the same as the fit of the measurement model (ΔCFI = .000, ΔTLI = .000, ΔRMSEA = .000). This implies that the non-modeled paths (those from SPSRQ factors to gambling behavior) were trivial. This model is displayed in Fig 1.

For the relations between SPSRQ and gambling motives, and restricting our attention only to the statistically significant coefficients (*p* < .05), Punishment sensitivity was positively related to Affect regulation, β = .28. Reward sensitivity was positively related to Fun/thrill, β = .36, Affect regulation, β = .22, and Social motives, β = .25. Regarding the relation between motives and gambling behavior, and describing only coefficients statistically significant and above |.30|, increments in Fun/thrill motives were associated with increments in frequency participation in Type I games, β = .56; increments in Affect regulation motives were related to increments in SOGS gambling severity, β = .59; Financial motives presented a positive relation with frequency of participation in Type II games, β = .36; and Social motives were negatively related to SOGS severity scores, β = –.48.

## Discussion

### The structure of gambling motives

The first aim of the study was to reach an integration of the factor structure underlying the (currently more widely used) gambling motives questionnaires. RGQ and GMQ-F have slightly different compositions, and the data-driven questionnaire resulting from our combination of both can help understand how specific motives relate to each other.

As described in our theoretical rationale, the most intuitive way to group motives together is to distinguish between positive and negative reinforcers. Yet, our results show this separation (on which both the GMQ-F and RGQ rely) might be exceedingly simplistic. Negative reinforcement (coping motives) allows gamblers to deal with negative affect, but some positive reinforcers, typically considered enhancement motives (e.g., “*I gamble because it makes me feel good*”) are also instances of affect modulation. Thus, as shown in the present study, when the valence-based distinction is not forced by confirmatory analyses, items of both valences tend to load onto a common factor. An inspection of such factor in the 4-factor solution is fully compatible with the interpretation that as many as eight items in the combination of RGQ and GMQ-F are clear markers of the use of gambling as an overt affect regulation strategy, independently of whether items are positively or negatively worded (three and five items, respectively). Other positive motives (Social, Financial, and Fun/thrill) are not necessarily linked to affect modulation, and can be factored apart from it.

This factor structure is compatible with the one reported in a study using principal-component analyses, and with the largest sample so far in gambling motives research [15]. A recent study [63] has successfully validated the Spanish version of the GMQ, in which enhancement and coping motives remain separated (with .85 and .83 internal reliabilities, respectively). Still, the correlation they reported between the two factors was *r* = .49, and both motives were similarly associated with gambling severity and signs of emotion/mood disorders. Similar patterns have been reported with the French version of the GMQ-F scale [16, 64]. In our study, some enhancement-related items from the GMQ and RGQ load in the Affect regulation factor, whereas others load in the Fun/thrill factor. In other words, the Fun/thrill factor inherits some items from the Recreational motives factor (e.g., “*As a hobby or pastime*”) from the RGQ, and some other from the Enhancement factor that are not clearly related to affect upregulation (e.g., “*Because it is exciting*”).

Beyond the theoretical value of these analyses, the second aim of the present study was to develop a brief and usable instrument to assess gambling motivation in Spanish speakers. The 18-item bGMI has a clearly identifiable and theoretically sound factor composition, with good internal reliabilities for the subscales that have been traditionally considered the most relevant in practical terms (Fun/thrill as a marker of gambling preferences and Affect regulation as a marker of severity). Reliability was lower for social motives but, still, this factor was significantly predicted by reward sensitivity, and was a strong indicator of reduced disordered gambling symptoms. Although additional research is however necessary to further ascertain the psychometric properties of the bGMI in clinical and non-clinical samples, it is also important to mention that the use of latent variables for motives dimensions in ESEM is explicitly intended to palliate measurement error problems in item-based scores.

### From gambling motives to gambling behavior

The third aim was exploring the role of gambling motives in the relationship between root personality dimensions and gambling behavior (severity and frequency). Confirming our hypotheses with regard to the second half of this pattern of links, the Affect regulation factor emerged as the strongest predictor of SOGS gambling severity in the model, whereas Social motives factor were negatively associated with gambling symptoms severity. No other motives were significantly related to gambling symptoms severity.

These results are consistent with previous studies, in which gambling to cope is associated with severity of disordered gambling symptoms [9, 16, 63, 64]. A representative study conducted with 2,121 participants [1], for example, found escape motives (mostly equivalent to coping motives) to be the best predictor of gambling problems, followed by excitement motives, and only more weakly by ego improvement motives. This study also confirmed that these relations were not mediated by optimism regarding the possibility to win money. In our study, however, the factor labelled as Fun/thrill did not predict disordered gambling symptoms, which supports the view that only some of the items included in the RGQ/GMQ Enhancement factor and in the excitement and ego improvement motives in the abovementioned study (i.e., those related to upregulation of general affect) are responsible for accounting for its relationship with problem gambling. Others, regarding fun, joy, or entertainment are surely less problematic.

Converging evidence shows the pivotal role of affect regulation in GD [65, 66, 67, 68]. More specifically, recent theoretical models–the *Gambling Space Model* [38] and the *Process Model*, [68], see also [69]–specify how altered affect regulation can fuel problem gambling at different levels. The present study portrays evidence that the different ways in which gambling can be used as an overt emotion regulation strategy are somewhat bounded together. Complementary evidence suggests that *covert* affect regulation strategies (e.g., reappraisal, refocusing) are used by gamblers to manage their desire to persevere in gambling behaviors in face of accruing losses, by means of the elaboration of complex cognitive distortions [65, 70]. The *Gambling Space Model* is indeed unique in its attempt to conjointly consider the roles of overt regulation, covert model-based (intentional) regulation, and covert model-free (incidental) regulation in an unified framework formulated to account for individual differences in gambling behaviors and gamblers clustering.

Social motives have been defined as gambling for social contact, to escape loneliness or isolation, for conformity, or to build family or community connections [41]. With regard to them, the present study yields a strong negative association with problem gambling symptoms (and a weaker one with gambling frequency for Type I and Type II games). This result aligns with previous literature that confers a protective role to social motives in gambling [27] and other putative addictive behaviors like video gaming [71, 72], or binge watching [73]. In other studies, social motives have been observed to be positively associated with problem gambling [19], although a closer look on these positive associations reveals that social motives are the weakest motive-related predictors of problem gambling [43]. These observations are consistent with the predictions of the *Pathways Model* [74], according to which social motives contribute to the pathway conducing to the *behaviorally conditioned* cluster of gamblers. This pathway is suggested to be less problematic and predicts a better prognosis, compared with the other two (conducing to *emotionally vulnerable* and *antisocial/impulsivist* clusters).

Regarding gambling frequency, the most salient association involves Fun/thrill motives and Type I gambling. This observation reinforces previous findings linking high-arousal, skill games with positive motives, and with traits that make individuals more prone to seek certain types of rewards [49, 75]. The remaining associations are weaker or non-significant, with the exception of the one between Financial motives and Type II gambling, which is consistent with previous reports showing this motive to relate with gambling frequency [41], and, most importantly, with symptoms of problem gambling associated with the use of electronic gaming machines [76], lotteries, and scratch cards [16], all of which are considered Type II games in [49] and in the present work.

### The personality roots of gambling motives and behavior

With regard to the role of reward and punishment sensitivity in gambling frequency and severity, previous evidence is mixed, in such a way that both hyper- and hyposensitivity to reward and punishment have been associated with problematic gambling. Reward sensitivity predicts gambling onset and signals disordered gambling [77]. But, at the same time, the role of gambling in compensating reduced effectiveness of natural rewards has also been suggested, on the basis of reports of blunted reward sensitivity in patients with GD [78, 79]. Similarly, individuals with low punishment sensitivity tend to gamble more frequently–probably due to diminished risk aversion [37], and patients with GD are less sensitive than controls to negative feedback in laboratory tasks [39]. Emotionally vulnerable gamblers, however, seem to present heightened punishment sensitivity scores, which renders them more vulnerable to problem gambling, via negative reinforcement [33, 80].

In line with the study by Wardell et al. [33], we found that individual differences in reward and punishment sensitivity predict gambling motives. Indeed, and despite the fact that the factorial structure of gambling motives in the two studies is not identical, their results are consistent. On the one hand, Reward sensitivity is associated with social and positive-reinforcement-related motives (in both instruments), but also with coping (RGQ) and Affect regulation motives (bGMI) (financial motives were not explicitly assessed in the RGQ version used in Wardell et al.’s study). On the other hand, Punishment sensitivity is associated with the Affect regulation factor in our model, and with Coping in Wardell et al.’s (which aligns with the view that gambling can be used to cope with anxiety and other aversive emotions associated with punishment sensitivity and neuroticism [81, 82]). The only major difference between the two studies is that, in ours, Punishment sensitivity was not associated with Fun/thrill motives, whereas in Wardell et al.’s study punishment sensitivity and enhancement motives weakly but significantly correlated. This difference portrays by itself some evidence that the Fun/thrill factor found in the present study does not entirely overlap with the Enhancement factor reported in previous studies.

Crucially, the fit obtained for the structural model is identical to the one of the measurement model, which implies that the removed paths were trivial. Although the present study is cross-sectional, and causal directionality cannot be established, the lack of direct links between Reward and Punishment sensitivity and gambling behavior is consistent with the mediational role of distinct motive types in the relationships between general psychobiological systems of reward and punishment-driven motivation and symptoms of problematic gambling.

### Practical implications

The association between coping motives and risk of gambling problems is one of the most solid and thoroughly discussed results in gambling clinical research. Although, as discussed earlier, reward-related motives have also been associated with gambling problems, the clinical implications of this relationship have remained underexplored. Our results, along with the ones of previous studies (e.g., [15]) suggest that, among appetitive motives, those not directly related to specific rewards (winning money, having fun, socializing), but to more diffusely improving mood, seem to be associated with clinically relevant features. This result resonates with models that attribute risk-signaling value to ego-syntonic motives for high engagement in putatively addictive activities (e.g., video gaming for self-esteem and self-identity [83]).

In general, individuals are motivated to maintain a positive mood [84], and gambling has effects on moods for some groups of people [85]. In our view, regularly resorting to gambling as a mood maintenance strategy could be indicative of a lack of alternative means to the same end, and thus deserves attention as a risk factor in preventive strategies that has remained mostly unnoticed in the past.

Apart from the link between Affect regulation motives and SOGS severity, some practical consequences can also be derived from the links between motives and gambling frequency for Type I and Type II modalities. According to the present results, frequency of participation in Type I games (casino, skills, cards, betting) is strongly driven by Fun/thrill motives, whereas participation in Type II games is motivated by a mixture of Financial, Fun/thrill, and Affect regulation reasons (in that order of importance). The association between Affect regulation motives and Type II gambling is congruent with the well-known high prevalence of problem gambling among slot machine gamblers in Spain [86]. However, most emerging gambling modalities in Spain, as in other parts of the world, are Type I, and understanding the kind of motives that drives new gamblers towards these new gaming activities is an important element of gambling marketing. Actually, exploiting Fun/thrill motives is a generalized advertising strategy to attract customers towards poker and betting sites [87], that has rapidly increased in the last years [88]. Appealing to these reasons has probably raised interest towards these forms of gambling in people who are more sensitive to them, namely youngsters (including underage ones) [89, 90].

### Concluding remarks, strengths and limitations

Our results did not fully support the five-factor solution that would stem from the mere summation of the GMQ-F and RGQ scales, nor the hypotheses derived from it. There seems to be no clear-cut separation between positive and negative reinforcement-driven motives, and an Affect regulation factor (including positively and negatively valenced items) emerged as the only one directly associated with severity of gambling symptoms, which largely replicates Lloyd et al.’s [15] findings. In accordance with this dual composition, the Affect regulation motives factor appears to be associated both with reward and punishment sensitivity.

These results must be considered in light of three main limitations. The first one, as noted above, is the impossibility to draw causal conclusions from mere sets of statistical dependencies. However, the vanishing of substantial associations of reward and punishment sensitivity with gambling problems, once conditioned on motives, is strongly indicative of the non-existence of direct causal links between SPSRQ dimensions and gambling frequency/severity, in any of the two directions [91]. Reward and punishment sensitivities are neurobiologically determined temperamental traits, but they are likely to interact with personal gambling history, exposure, or specific game design features to end up shaping gambling behavior and potential problems resulting from it.

Secondly, our sample size was relatively small. This has two effects: (a) although our dataset was sufficiently large to yield an interpretable covariance matrix, it did not allow carrying out analyses for specific subsets of participants; and (b) our results may not be stable even with new samples with the same sampling approach due to random error.

And thirdly, sampling was intended to include gamblers across the whole severity continuum, and, thus, a proportion of disordered gamblers was included to ensure a sufficient number of observations at pathological levels. Convenience, non-random samples with non-proportional numbers of individuals in high or low levels of the severity continuum (relative to what is common in the population of reference) is pervasive in the literature (see, for example [10, 51, 92, 93, 94]). Unavoidably, this method generates samples that are not closely representative of any particular demographic group. Still, it is also the only way to avoid problems as the one previously described for the original RGQ validation [21], while retaining a large majority of non-problem gamblers in the sample. The combination of a small sample size with a non-representative sample implies that the results may reflect sample characteristics. Small samples sizes imply large standard errors in our estimates and non-representative samples lead to unknown bias in the parameters. Our main interest was to relate gambling motives with BIS/BAS and gambling behavior and severity. Our observations that (a) BIS/BAS effects on gambling behavior are likely to be mediated by specific gambling motives, and (b) that the motives related to regulation of affect are more likely to be indicative of gambling problems, are valuable by themselves, but have to be considered in light of our sample limitations. Thus, although our methods seem adequate to provide a general view of the structure, precursors, and manifestations of gambling motivation, we cannot guarantee that such structure and relations will remain invariant in other samples or across subpopulations of gamblers within different ranges of severity or different demographic groups. Future studies are encouraged to try to cross-validate our results with larger and more representative samples, and with specific subpopulations.

A first strength of the present study is its data-driven approach. Indeed, we have remained open to the possibility that the data did not support our initially preferred theoretical framework (as it actually happened). Secondly, relying on latent variables rather than manifested ones increases the reliability of construct measurement, and reduces the risk that results are affected by measurement error [95]. And finally, the combination of items from the two dominant models allowed us to begin with a large and diverse set of motives that are likely to be representative of most motives gamblers would freely report [48]. From these, it was possible to select the most discriminative ones, in order to build a usable gambling motives inventory to be implemented in future research and clinical practice.

## Appendix. Spanish items as presented to the participants in the current study

1. Porque me preocupa no ganar si no juego

2. Para competir con otros (p.e. corredores de apuestas, otros jugadores)

3. Porque disfruto pensando en lo que haría si ganase el premio gordo/el bote

**4. Para conseguir ingresos**

**5. Como una afición o pasatiempo**

6. Para evitar el aburrimiento o matar el tiempo / pasar el rato

**7. Porque es divertido**

8. Porque es algo que hago con mis amigos o familia

**9. Porque me siento más confiado/a y seguro de mí mismo/a**

**10. Porque es lo que la mayoría de mis amigos hacen cuando nos reunimos**

11. Porque me siento realizado cuando gano

**12. Porque es emocionante**

**13. Para relajarme**

14. Por el desafío mental o para aprender sobre el juego o actividad

**15. Porque ganando cambiaría mi estilo de vida**

**16. Para animarme cuando estoy de mal humor**

**17. Para olvidar las preocupaciones**

**18. Para ganar dinero**

**19. Porque me ayuda cuando estoy tenso**

20. Para relacionarme con los demás

**21. Porque es algo que hago en ocasiones especiales**

**22. Porque hace las reuniones sociales más divertidas**

23. Para impresionar a otras personas

**24. Porque me hace sentir bien**

**25. Porque me gusta cómo me hace sentir**

**26. Por la posibilidad de ganar mucho dinero**

**27. Porque me ayuda cuando me siento nervioso o deprimido**

Response options and scoring:

Casi nunca/Nunca = 0

A veces = 1

A menudo = 2

Casi siempre/Siempre = 3

Note: Items in bold were included in the brief Gambling Motives Inventory (bGMI). Items are presented in the same order as in Table 1.
